# Smoking Cessation Induces Profound Changes in the Composition of the Intestinal Microbiota in Humans

**DOI:** 10.1371/journal.pone.0059260

**Published:** 2013-03-14

**Authors:** Luc Biedermann, Jonas Zeitz, Jessica Mwinyi, Eveline Sutter-Minder, Ateequr Rehman, Stephan J. Ott, Claudia Steurer-Stey, Anja Frei, Pascal Frei, Michael Scharl, Martin J. Loessner, Stephan R. Vavricka, Michael Fried, Stefan Schreiber, Markus Schuppler, Gerhard Rogler

**Affiliations:** 1 Division of Gastroenterology and Hepatology, University Hospital Zurich, Zurich, Switzerland; 2 Division of Internal Medicine, University Hospital Zurich, Zurich, Switzerland; 3 Division of Clinical Pharmacology and Toxicology, University Hospital Zurich, Zurich, Switzerland; 4 Institute of Food, Nutrition and Health, ETH Zurich, Zurich, Switzerland; 5 Institute of Clinical Molecular Biology, Christian Albrechts University of Kiel, Kiel, Germany; 6 Division of General Internal Medicine, University Hospital Schleswig-Holstein, Kiel, Germany; 7 Institute of General Practice, University of Zurich, Zurich, Switzerland; 8 Smoking Consulting Programme, University Hospital Zurich, Zurich, Switzerland; 9 Division of Gastroenterology and Hepatology, Hospital Triemli, Zurich, Switzerland; Charité, Campus Benjamin Franklin, Germany

## Abstract

**Background:**

The human intestinal microbiota is a crucial factor in the pathogenesis of various diseases, such as metabolic syndrome or inflammatory bowel disease (IBD). Yet, knowledge about the role of environmental factors such as smoking (which is known to influence theses aforementioned disease states) on the complex microbial composition is sparse. We aimed to investigate the role of smoking cessation on intestinal microbial composition in 10 healthy smoking subjects undergoing controlled smoking cessation.

**Methods:**

During the observational period of 9 weeks repetitive stool samples were collected. Based on abundance of 16S rRNA genes bacterial composition was analysed and compared to 10 control subjects (5 continuing smokers and 5 non-smokers) by means of Terminal Restriction Fragment Length Polymorphism analysis and high-throughput sequencing.

**Results:**

Profound shifts in the microbial composition after smoking cessation were observed with an increase of *Firmicutes* and *Actinobacteria* and a lower proportion of *Bacteroidetes* and *Proteobacteria* on the phylum level. In addition, after smoking cessation there was an increase in microbial diversity.

**Conclusions:**

These results indicate that smoking is an environmental factor modulating the composition of human gut microbiota. The observed changes after smoking cessation revealed to be similar to the previously reported differences in obese compared to lean humans and mice respectively, suggesting a potential pathogenetic link between weight gain and smoking cessation. In addition they give rise to a potential association of smoking status and the course of IBD.

## Introduction

The human intestinal microbiota has important influences on the development of innate immunity [1], [2], regulation of epithelial development and nutrition [3], [4]. The gut microbiota and alterations in its complex composition have been identified as an contributing factor in the pathogenesis of various diseases, such as inflammatory bowel disease (IBD) [5], [6] or irritable bowel syndrome [7]–[9]. Cigarette smoking is considered to be one of the most important environmental risk factors in IBD pathogenesis [10]–[13]. In the two main subtypes of IBD, Crohn's disease (CD) and ulcerative colitis (UC), there is a known divergent effect of smoking on the disease course. While smoking is clearly detrimental in CD [14], [15] (in many [16] but not all [14] studies this seems especially to be the case with regard to ileal CD) it has a well-known protective effect in UC with a lower incidence of the disease in smokers [11] and a more severe disease course after smoking cessation [17]. In addition, the gut microbiota seems to play a crucial role in the pathogenesis of obesity and the metabolic syndrome [18], [19], characterized by distinctive shifts in the relative abundance of mayor phyla in obese vs. lean humans [20], [21] and mice [22], respectively. Around 80% of individuals who cease smoking gain weight to an average of 7–8 kg [23], interestingly even despite stable [24] or even decreased [25] total caloric intake. Furthermore an alteration of the oropharyngeal and tracheal microbiota in smokers compared to non-smokers has been shown recently [26].

In the last few years considerable progress has been achieved in the comprehension of the enormous diversity of the intestinal microbiota, its component genes (microbiome) and host genetic factors influencing its development after birth with the spread of culture independent methods [27]. Nevertheless, the precise role of environmental factors, such as nutrition, medication use or smoking on the composition of the gut microbiota is largely unknown. Accordingly, and in view of the above mentioned microbial discoveries with regard to the pathogenesis of obesity and IBD we aimed to investigate the development of human intestinal microbial composition during controlled smoking cessation. Microbiota analyses of repetitive stool samples were performed in the course of a controlled prospective study with 10 healthy smoking subjects undergoing smoking cessation (intervention group) and 10 healthy control subjects, 5 continuous smoking (control group smokers) and 5 non-smoking (control group non-smokers) subjects. Our hypothesis at the beginning of the study was, that smoking may influence the composition of the intestinal microbiota and accordingly, that smoking cessation may also alter intestinal microbial composition. We further hypothesized, that the weight change after smoking cessation migt be associated with a shift to a microbiota pattern harbouring similarities to the recently characterized found in the “obese microbiota” in humans or animal models and that these microbial shifts might indicate a pattern associated with a pro-inflammatory situation.

## Materials and Methods

### Study design

The study was fully approved by the local Ethics Committee. Observation period was 9 weeks (1 week before and 8 weeks after cessation of smoking) including 5 study visits, among them t1 (Screening), t2 and t3 (4 and 8 weeks respectively after smoking cessation). All 5 study visits took place in facilities of the smoking consulting program of the University Hospital Zurich, Switzerland. At screening visit (S = t1) written informed consent was obtained. Inclusion (age ≥18 and ≤60 years; written consent; smoker (defined as daily cigarette consumption of ≥10); readiness to quit smoking) and exclusion criteria (intercurrent bacterial or viral disease of the colon; pregnancy, lactation; patients with more than 40 mg prednisone per day (or equivalent); diabetes mellitus; severe comorbid disease (making a participation at the study not possible according to opinion of the test physician); justified doubt about the co-operation of the patient; patients with active infection or systemic antibiotic, antiviral, or antifungal treatment in the last 3 weeks before the screening investigation; patients with alcohol or drug misuse in the last year; patients with short bowel syndrome; patients with parenteral nutrition; severe comorbid conditions) were checked. Full medical record, medication use, alcohol intake and smoking habits were assessed and a physical examination including body height and weight was performed. During the whole study period intensive counseling was provided by both, physicians and psychologists with special experience in consultation in smoking cessation. Subjects were instructed to keep on smoking according to their regular smoking habits up to baseline (T0; 7–10 days after screening). Baseline was prespecified as the date of complete smoking cessation.

At the baseline study visit and the subsequent control visits 1 week (T1), 4 weeks (T4) and 8 weeks (T8) after smoking cessation physical examination including body weight and assessment of medication intake was re-performed. Mandatory strict adherence to complete smoking cessation was verified by a breath carbon monoxide monitor (piCO+TM Smokerlyzer®; Bedfont Scientific Ltd & decode.uk). Measuring was done in concordance to the guidance of the manufacturer, always performed under direct observation of a study physician. A cut-off level of >6 ppm was chosen to define current smoking [28]; a single level above 6 ppm or self-declaration of on-going smoking after T0 led to study drop out. Supplemental nicotine administration or use of smoking cessation medications was not allowed. Stool samples, which have been shown to be representative of inter-individual differences in microbial composition [29], were collected at every study visit. Samples of 3 time points were used for further analyses; at screening (one week prior to smoking cessation, t1), 4 and 8 weeks after smoking cessation (t2, t3) corresponding to week 0, 5 and 9 in the continuing smoking and non-smoking subjects. Study subjects had to complete a daily food-frequency protocol three times over a period of 7 consecutive days (one week before t1, t2, and t3), allowing assessment of food patterns in a qualitative and approximated quantitative manner.

### Microbiota analyses

Stool samples were collected in conventional fecal samples storage tubes containing phosphate buffered saline (PBS)/Ethanol, the latter leading to an immediate denaturation of bacterial cell wall. Methods for T-RFLP are depicted in the SI Materials and Methods. For pyrosequencing genomic DNA from fecal pellets was extracted and quantified. We amplified variable region V1–V2 of the 16S rRNA gene using forward and reverse primer, the latter containing a unique 10 base multiplex identifier (MIDs designated as XXXXXXXXXX) to tag each Polymerase Chain Reaction (PCR) product. Replicate PCRs were performed for each sample and negative controls. The amplified product was run on agarose gel and the specific band was excised and amplicons were purified. Equal amount of PCR products were mixed in a single tube and sequenced using Roche® 454 titanium chemistry. Replicate PCRs were performed for each sample as well as negative controls (water). All sequence reads were screened and filtered for quality and length; chimeric sequences were removed as were sequences failing quality criteria (further details in the SI Materials and Methods). Taxonomy was assigned by comparison against the Ribosomal Database Project (RDP) release 10 [30]. The complete raw sequencing data of this study is accessible via the European Nucleotide Archive in the Sequence Read Archive (SRA) under the study accession number ERP002222 (available at the URL http://www.ebi.ac.uk/ena/data/view/ERP002222; Samples accessions: ERS212677).

### Statistical Analysis

Paired Student's t-test was used for a comparison of average daily calorie intake (and respective fraction of calories attributable to carbohydrates, proteins, fat, fibres and alcohol). Analyses of the T-RFLP data was performed using T-Rex (T-RFLP analysis Expedited) [31]. A doubly centred Principal Component Analyses (PCA), also known as the Additive Main Effects and Multiplicative Interaction Model (AMMI) with 9 environments (intervention group, control group smokers, control group non-smokers; each at t1, t2 and t3) was performed. For the sequences obtained by pyrosequencing paired Student's t-test was used for taxonomy based comparison of between group changes. Beforehand skewness of value distribution was ruled out with StatView™. Cd-hit [32] was used to identify and cluster similar sequences for phylogenetic analyses and to define OTUs with the commonly applied sequence identity cut-off of 0.97. Sequence alignment was performed using NAST from the Greengenes server [33] including all sequences with a minimum length of 150 bases. A phylogenetic tree was constructed using FastTree 2. To perform a phylogeny-based analyses of beta diversity we used Fast UniFrac [34], a bioinformatic software tool that can be used to characterize variations in bacterial community membership over time in dependency of external influences, such as nutrition or space in a large numbers of samples and sequences. Analyses in Fast UniFrac can be performed either by considering only the absolute abundance of sequences in every sample (unweighted) or by taking the relative abundance of bacterial species into account (here, the branches of certain bacterial lineages are weighted according to their quantitative occurrence). A normalization step can be performed (each sample treated equally instead of each unit of branch as in the non-normalized mode; for normalization the UniFrac value is divided by the distance scale factor, which is the average distance from the root of each sequence). Initially, sequences were processed by identifying and selecting the occurring OTU and a phylogenetic tree was built. A sample distance matrix was constructed, showing the overall phylogenetic distance between each pair of sequences. This matrix provides the basis to conduct PCA and hierarchical clustering using the UPGMA (Unweighted Pair Group Method with Arithmetic Mean) algorithm.

Bonferroni correction was applied in case of multiple testing. As a resampling method bootstrapping was performed including 75% of available sequences in each run in altogether 1000 permutations using the software tool QIIME [35] (Quantitative Insights Into Microbial Ecology).

The term phylogenetic diversity was coined by Faith [36] and characterizes the amount of biodiversity in a given sample by summarizing the total length of all branches leading to the investigated set of taxa in a phylogenetic tree, i.e. a quantification of the phylogenetic heritage of organisms in a given set of species. Alpha diversity was calculated using the Phylogenetic Diversity Analyser (PDA) [37]. Rarefaction curves were constructed using QIIME.

## Results

### Calorie intake and body weight in the course of smoking cessation

During the observational period there was a mean 2.2 kg increase in body weight in the 10 subjects undergoing smoking cessation (mean body weight at screening 71.8 kg, corresponding to a mean BMI of 24.1 kg/m^2^; mean body weight at T8 74.0 kg, corresponding to a mean BMI of 24.8 kg/m^2^; Table S1). However, neither a significant alteration of total average daily calorie intake, nor the fraction of nutritional components (carbohydrates, proteins, fat, fiber and alcohol) was identified based on the weekly food-frequency-protocols as indicated in Figure S1 and Tables S2, S3 (as a sole exception a slight but significant increase in calorie intake from alcohol between t2 and t3 was observed).

### Alteration in the microbial composition, indicated by T-RFLP analysis

As a first step we conducted a universal terminal-restriction fragment length polymorphisms (T-RFLP) analysis. Doubly centered Principal Component Analyses (PCA) of the resulting data with 9 environments (intervention group, control group smokers, control group non-smokers; each at t1, t2 and t3) indicated a clear shift in the intervention group between samples from t1 and t2, as well as t2 and t3. The observed shift was most pronounced along the first principal component axis, representing the major part of the observed difference (58.5%; Figure S2). No such shift was observed in the non-smoking control group, where a close clustering was maintained over the different time points. In the smoking control group a somewhat wider separation was detected, however almost exclusively along the second principal component axis. The latter represents substantial less difference in species composition (37.7%) as compared to the first principal component axis (Figure S2, Table S4).

### Differences in the abundance of sequences before and after smoking cessation

For a more comprehensive analysis of the microbial diversity harbored by the stool samples we next performed a 454 pyrosequencing approach to analyze the variable regions V1–V2 of the 16S rRNA gene. After applying an initial cleaning step on the generated raw sequence data as described in the methods section we generated a total of 335′902 sequences. The vast majority of sequences (97.3%) could be assigned to four phyla: *Firmicutes* (51.9%), *Proteobacteria* (33%), *Bacteroidetes* (7.1%) and *Actinobacteria* (5.4%) (Further identified bacteria included members of the phyla *Verrucomicrobia*, *Lentisphaerae*, *Tenericutes*, *Cyanobacteria*, *Synergistetes*, *Fusobacteria*, *Deinococcus-Thermus*, *TM7*, *Acidobacteria* and *OD1*, whereas 0.9% of sequences could not be classified (Figure S3)).

**Figure 1:**
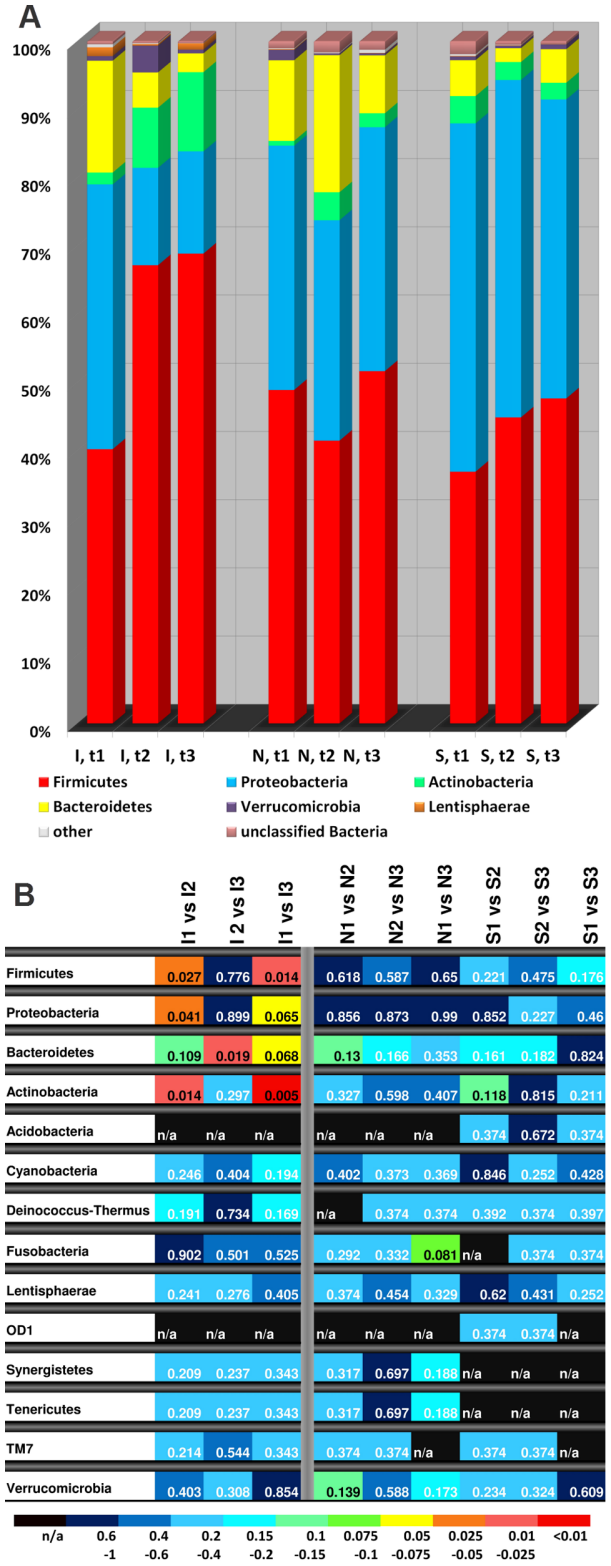
Comparison of the major phyla of the gut microbiota before and after smoking cessation. (A) Phyla Composition. The results for the intervention group (I) and the control groups (non-smoking  = N; smoking  = S) are given for samples taken one week prior to smoking cessation (t1) as well as four weeks (t2) and eight weeks (t3) thereafter. Whereas the intervention group revealed a significant increase in fractions of *Firmicutes* and *Actinobacteria* and a decrease in fractions of *Proteobacteria* and *Bacteroidetes*, the microbiota of the control groups remained rather stable. The phyla *Tenericutes*, *Verrucomicrobia*, *Synergistetes*, *Fusobacteria*, *Deinococcus-Thermus*, *TM7*, *Acidobacteria* and *OD1* are summarized under “other”. (B) Heat Map. The result of paired Student's t-test is shown on the phylum level with a color coded heat map. Significance levels are shown in different colors (shades of red, significant shifts in bacteria composition; shades of yellow, green and blue, non-significant shifts) and are indicated by the exact significance values within the colored squares of the graph. The major changes in the microbiota in the intervention group were observed between the time points before (t1) and after (t2, t3) smoking cessation. In contrast no significant changes were detected in the control groups and – with the exception of *Bacteroidetes* – after smoking cessation between t2 and t3 in the intervention group (an extended version of the heat map including all identified genera is shown in Figure S5, n/a =  not applicable).

We found an increase of sequences from *Firmicutes* and *Actinobacteria* and a simultaneous decrease of the *Proteobacteria* and *Bacteroidetes* fractions after smoking cessation (Figure 1a). These changes were exclusively observed in the intervention group and a substantial part of the shift occurred between t1 and t2. The increase of *Firmicutes* and *Actinobacteria* as well as the decrease of *Proteobacteria* between t1 and t2 in the intervention group was significant in the paired Student's T-Test (0.027, 0.014 and 0.041 respectively), while the decrease in the fraction of *Bacteroidetes* did not reach statistical significance (0.109). Although the detected changes between t1 and t2 seemed to be further accentuated 8 weeks after smoking cessation in t3 (except for the *Proteobacteria*) the composition of phyla between t2 and t3 remained strikingly similar with the exception of *Bacteroidetes*, pointing to both a relatively brisk (within 4 weeks) and durable (8-week interval) effect of smoking cessation on microbial composition. In contrast, the composition of phyla in both control groups remained relatively stable. Here not one single change in the composition of phyla reached statistical significance (Figure 1b). However, the observed changes in the control groups were not exclusively restricted to the phylum level. Significant changes were also detected on the genus level (Figure S5). We did not observe any per individual correlation between changes in body weight and microbial composition.

### Differences in microbiota composition indicated by phylogenetic microbial analyses

**Figure 2:**
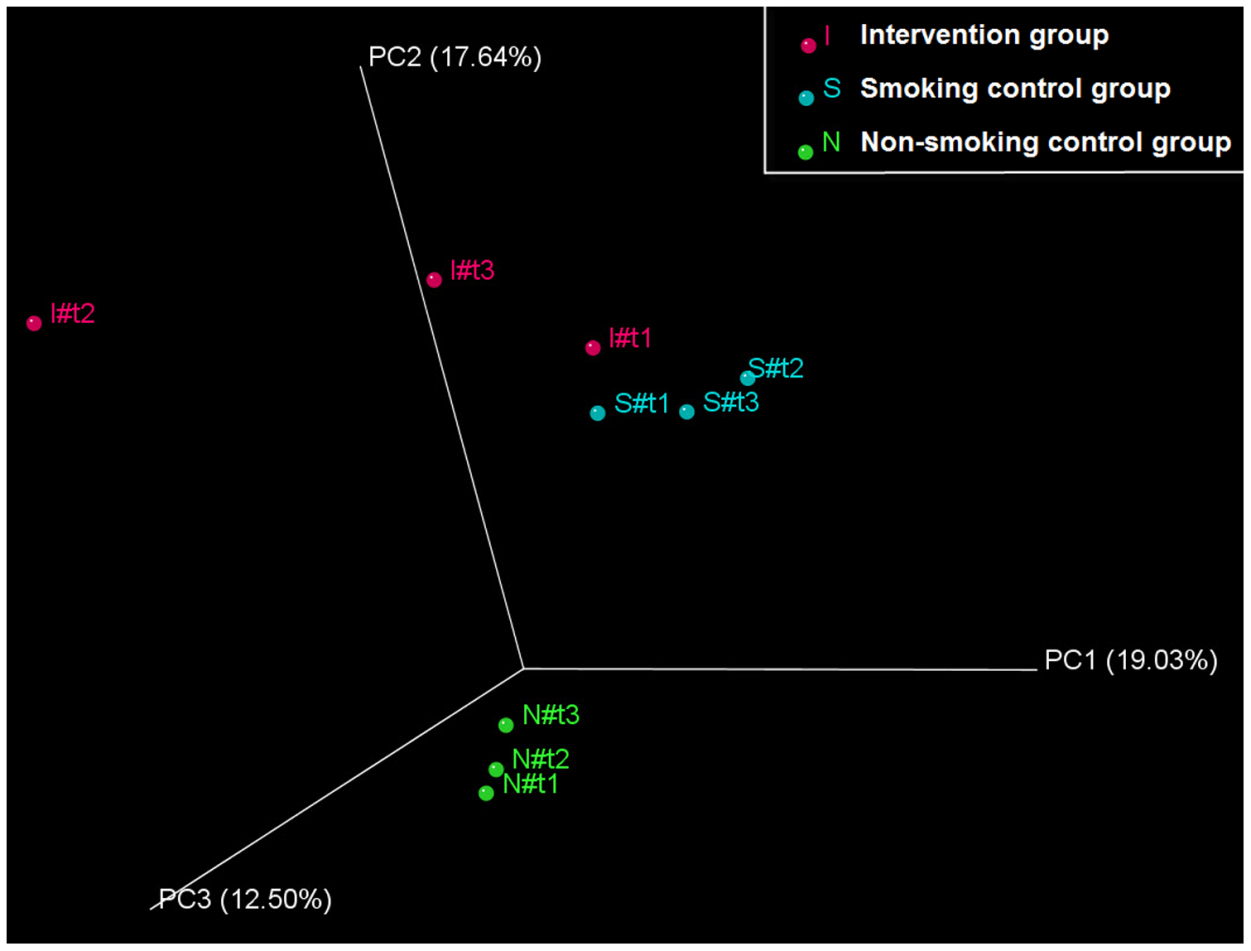
Phylogeny-based Principal Component Analysis. Bacterial communities of the three different treatment groups were clustered using PCA and the unweighted UniFrac distance matrix as an input (weighted PCA is shown in Figure S6). With PCA, a multivariate statistical analyses, axes that reflect the largest part of sample variation are identified (Percentage values at the axes reflect the level of variation explained by each principal coordinate; the first axis indicates the largest fraction of difference). Separation of the different sample collectives in 3 dimensions is visualized. A separation of the samples from the intervention group (I), that is most predominant 4 weeks after smoking cessation, was revealed. In contrast, the samples from the non-smoking (N) and smoking (S) control groups clustered together closely, thus reflecting their overall similar microbial composition.

**Figure 3:**
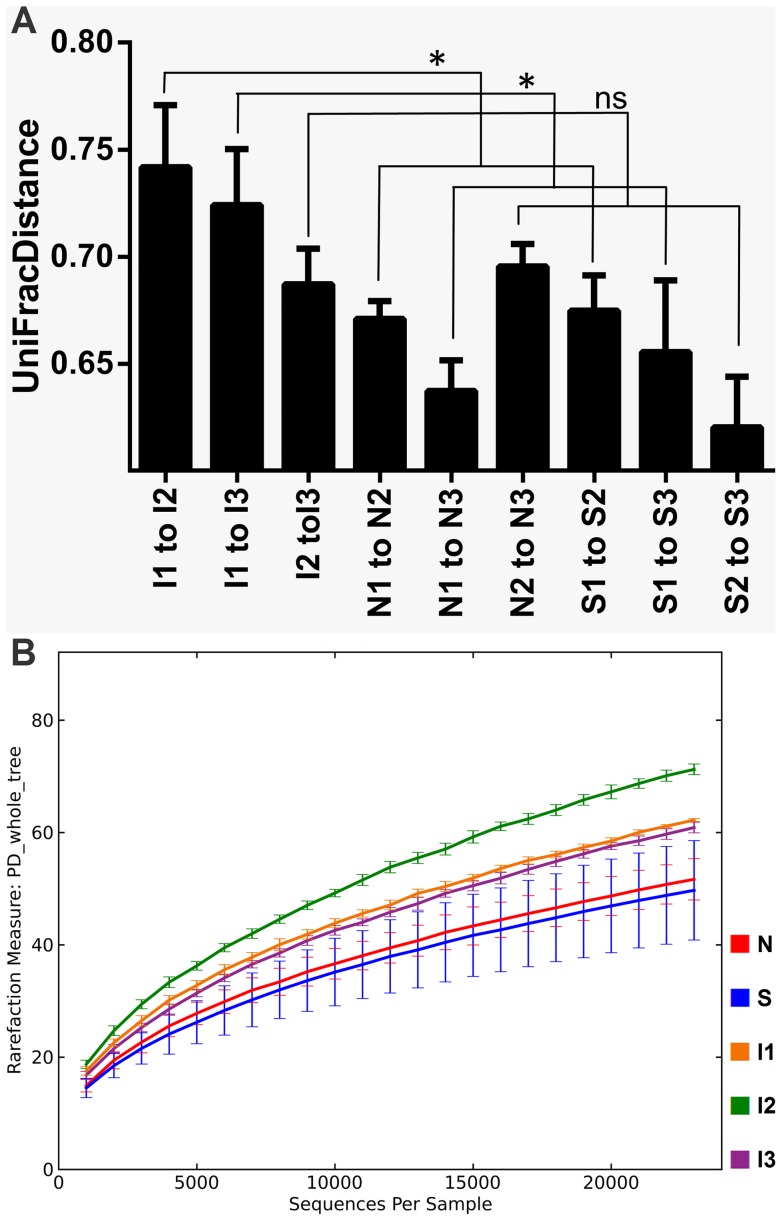
UniFrac distance between samples and rarefaction curve of phylogenetic diversity. (A) Unweighted UniFrac distance. The higher a UniFrac distance value between two samples the more different the bacterial composition. The highest distance values were determined for subjects undergoing smoking cessation between t1 and t2 as well as between t1 and t3. All other distance values were substantially smaller (error bars indicate SEM; *: p<0.05; ns: not significant). (B) Rarefaction curves. These curves express the accumulation of phylogenetic richness that would be obtained with continuous sampling effort and hence minimize potential differences that would be a result of the variable number of sequences obtained per sample. For the control groups the three sampling time points were combined in a single curve, while for the intervention group separated curves for t1, t2 and t3 were depicted to visualize the increased phlyogenetic diversity (PD) in the samples 4 weeks after smoking cessation (I2) compared to I1 and I3 (additional indices of α-diversity are depicted in Figure S9).

PCA including 9 environments revealed a clear separation of bacterial community composition in the intervention group, most pronounced between t1 and t2, whereas the control groups clustered closely together (Figure 2 and Figure S6 for unweighted and weighted analyses, respectively). UniFrac distance serves as a measure of difference in the phylogenetic lineages between different environments. The highest UniFrac distance was determined in subjects undergoing smoking cessation, comparing the time points prior to and after the intervention (I1 to I2 versus N1 to N2 and S1 to S2: p = 0.045; I1 to I3 versus N1 to N3 and S1 to S3: p = 0.026). In contrast no significant differences between the samples from the intervention and control groups were observed after the intervention (I2 to I3 versus N2 to N3 and S2 to S3: p = 0.244, not significant). These findings illustrate that the samples prior to and after smoking cessation were more different to each other (Figure 3a). In addition, clustering the samples based on the distance matrix the separation of bacterial lineages at t1 and t2 in the intervention group occurred significantly closer to the root (i.e. higher UniFrac distance units) of the constructed tree compared to any other separation process observed within intervention and control groups. Weighted UniFrac Significance reflected a significant difference between t1 and t2 (non-normalized UniFrac Significance 0.036, with Bonferroni correction applied). After normalizing, significance was additionally observed for t1 to t3, exclusively in the intervention group (<0.001 for both, t1 to t2 and t1 to t3, Bonferroni correction applied). Jackknife analysis used as a statistical technique of repetitive resampling (bootstrapping) revealed perfect reproducibility underlining robustness of our results (Figure S7).

### Microbial diversity analyses (α-diversity)

Both Phylogenetic Diversity and endemic Phylogenetic Diversity [38] (the sum of all branch lengths of species exclusively found in the respective environment) as measures for α-diversity were shown to be substantially higher 4 weeks after smoking cessation compared to the samples obtained whilst smoking. After 8 weeks there was still a trend towards increased diversity levels compared to baseline. In the control groups both diversity indices were relatively stable during the whole observation period (Figure S8). Rarefaction curves, a means of correction for number of sequences obtained per sample, underlined the increased microbial diversity after smoking cessation (Figure 3b, Figure S9).

## Discussion

The results of our controlled trial suggest that smoking is to be included in the growing list of known factors influencing the composition of the intestinal microbiota. We found evidence with two independent methodological approaches that smoking cessation induces a substantial modification of the intestinal microbial composition with no indication of any concomitant alteration in nutrition. A variety of different analyses of the high-throughput sequencing data indicated differences before and after smoking cessation, suggestive for profound and robust microbial shifts. On the phylum level strikingly even alterations regarding the major four phyla were identified in the individuals undergoing smoking cessation (Figure 1, Figure S4). On the level of OTUs quantitative changes (relative abundance of some bacterial lineages in the complex microbial mixture) are indicated by alterations in the weighted UniFrac mode, while the similar findings in the unweighted analyses in addition point to qualitative changes (i.e. occurrence of new OTUs after smoking cessation and disappearance of former OTUs prior to the intervention).

Furthermore, aside from pure microbial shifts an increase in microbial plurality is suggested by the increase in α-diversity after smoking cessation (Figure 3b, Figure S9). However, due to the limited observation period after smoking cessation, it is not possible to draw any conclusions on a potential sustained long-term increase of α-diversity due to smoking cessation. A long-term effect towards an increased microbial richness rather appears doubtful, as the initial increase in diversity somewhat mitigated after 8 weeks in all our analyses of α-diversity. Also, there were no substantial differences in α-diversity between smokers and non-smokers in the control groups. Interestingly, in contrast to our findings, oropharyngeal microbial diversity was found to be increased in smokers compared to non-smokers [26]. Thus, to clarify this issue further studies on the long-term effect of smoking on α-diversity of the intestinal microbiota are necessary.

We observed similar shifts in microbial composition on the phylum level after smoking cessation as described in obese versus lean humans [21], [20] and mice [22] (a lower proportion of *Bacteroidetes* and a higher proportion of *Firmicutes* and *Actinobacteria*), while a concomitant average weight gain of 2.2 kg during the whole observational period occurred. However, neither a change in total daily calorie intake nor nutritional components was suggested owing to analyses of the food-frequency questionnaires. Thus, the common perception that a change in dietary habits is the key factor for weight gain after smoking cessation is challenged. Our findings may suggest a potential role of an altered composition of the intestinal microbiome and hence metabolome with subsequent modifications in gut microbiota's metabolic function in the pathogenesis of weight gain after smoking cessation.

The above mentioned disordered oropharyngeal and tracheal microbial communities in smokers [26] appears to be intuitively plausible due to the direct contact of cigarette smoke with the respiratory epithelial cells and mucus harboring the local microbiota. Possible pathophysiologic links explaining the influence of smoking and smoking cessation on the intestinal microbial composition are much less evident. However, our study was not designed to investigate potential causative mechanisms. In addition, due to the study design it was not possible to investigate whether there are any loco-regional differences such as for instance between the terminal ileum and recto-sigmoid. This would be of interest with regard to the discrepancy of smoking between UC and CD.

Another limitation of our study is that despite the controlled study setup to largely rule out external influences such as for instance medication intake (e.g. antibiotics or supportive drugs for smoking cessation) or non-adherence to strict smoking cessation, we cannot completely ascertain lack of confounding effects such as exercise or subtle changes in dietary habits unrecognized by analysis of food-frequency protocols. However, the following considerations argue against a major contributing role of hidden dietary alterations. First, the assumption that an alteration of diet and increase of calorie intake would explain the observed weight gain is not based on robust evidence, as indications that increased calorie intake is the principal explanation for weight gain after smoking cessation are at best conflicting. In contrast, there are important studies suggesting alternative mechanisms for weight gain, such as a decreased metabolic resting rate [39] or changes in metabolic properties in the adipose tissue, in specific an increase of the lipoprotein lipase activity [40]. While a smaller study found no increase in calorie intake after smoking cessation in subjects gaining weight [24], an indicator for alternative mechanisms for the well-known effect of weight gain [23] are the results of the multiple risk factor intervention trial [25]; one of the largest studies ever performed in this field. This trial included more than 11000 men with an observational period of 6 years, thoroughly investigating weight gain and calorie intake in nonsmokers, quitters, recidivists, and continued smokers in a usual care group in comparison to a special intervention group with specific counseling. Interestingly, despite a reduced calorie intake and a generally healthier diet after counseling compared to baseline, and an even bigger reduction in calories compared to continuing smokers, men who ceased smoking gained weight. This was strikingly in contradiction to nonsmokers, continuing smokers, and even recidivists, who all lost weight. Second, although alterations in intestinal microbiota composition due to diet are well known, profound shifts seem to necessitate a longer time period as a recent human study found significant changes on the phylum level to occur predominantly after 12 weeks [21]. In contrast no stable switches between enterotype groups occurred in the short-term, despite a controlled and randomized short-term feeding experiment using two extremely divergent diets (high-fat/low-fiber versus low-fat/high-fiber) [41]. Third, a uniform shift in the phyla composition such as the one observed in our patients undergoing smoking cessation (Figure S4) indeed might be assumed to occur in interventions with prespecified and standardized major changes of diet. But this would less likely to be expected in individually different and uncontrolled slight dietary changes undetected by food-frequency protocols.

Although we identified continuous microbial shifts 8 weeks after smoking cessation, this relatively short observational period does not permit conclusions about a long-term alteration. Nevertheless, the continuing increase in body mass observed in subsequent 4 years [23] after the first year after smoking cessation (where the major part of the weight gain occurs) points to a potential sustained microbial alteration. Larger prospective long-term studies are needed to address this issue.

Although the role of cigarette smoking as one of the most important environmental risk factor in the pathogenesis of IBD is well established [10]–[13], the numerous epidemiological reports on smoking status and course of IBD are in sharp contrast with the very limited mechanistic explorations of the effect of smoking cigarettes on the gut and intestinal inflammation. The molecular and cellular mechanisms by which smoking interferes with the pathogenesis of CD and UC are only poorly understood. While several potential mechanisms, such as modulation of mucosal immune responses, alterations in intestinal cytokine and eicosanoid levels or modifications in gut permeability have been proposed, none of these hypotheses could hitherto offer a satisfying explanation [42]. As the importance of the intestinal microbiota in the pathogenesis of IBD is well established, the profound shifts in the composition of the intestinal microbiota observed in our study may shed some light to a potential pathogenetic link of cigarette smoking, smoking cessation, and course of disease in IBD, including the discrepancy in CD and UC. However, as we did not investigate the intestinal microbial evolution during smoking cessation specifically in patients with IBD, this potential pathogenetic link largely remains a speculative deduction and our findings need to be confirmed in CD and UC patients. We nevertheless believe, that the results of our observational study, demonstrating intestinal microbial shifts after smoking cessation for the first time, point to a potential interaction of disease-modifying factors in IBD. This may give us a further piece of information with regard to an enormously complex interplay, where we clearly are just at the beginning of our understanding – the interplay of the intestinal microbiota, chronic inflammatory bowel disease, systemic metabolic interactions and environmental factors (such as smoking).

In conclusion, smoking cessation induces profound changes in intestinal microbial composition. Interestingly, these changes appear to occur in a comparable manner both microbiologically and clinically (weight gain) as those induced by transplantation of an ‘obese microbiota’ into lean mice [20]. These alterations in conjunction with weight gain point to further evidence for the assumed role of the intestinal microbiota and its metabolic properties derived to the host in the pathogenesis of obesity and the metabolic syndrome. Our findings invite confirmatory larger and long-term studies as well as further research on the effect of environmental factors on intestinal microbial composition.

## Supporting Information

Figure S1 **Average daily calorie intake.** No significant changes in total calorie intake or nutritional components were observed before (t1) and after smoking cessation (t2, t3), except for a modest increase in average daily alcohol consumption between t2 and t3 (error bars indicating SEM; Carb: carbohydrate; da: daily average). Further details on the individual calorie intake are given Table S2 and S3.
(TIF)

Figure S2 **PCA with 9 environments.** Separation of the samples from the intervention group (E7 = I, t1; E8 = I, t2; E9 = I, t3) alongside the first principal component axis (IPCA1) reflecting the largest difference, 58.5% (as outlined in Table D, Table S4.). There is a close association of samples from the non-smoking control group (E1 = N, t1; E2 = N, t2; E3 = N, t3). A wider separation is to be seen in the smoking control group (E4 = S, t1; E5 = S, t2; E6 = S, t3), however almost exclusively alongside the second principal component axis (IPCA2), reflecting a substantially smaller difference (37.7%).
(TIF)

Figure S3 **Distribution of phyla in all samples in percentages.** The fraction other (0.1% of all sequences) in the right pie chart consists of *Synergistetes*, *Fusobacteria*, *Deinococcus-Thermus*, *TM7*, *Acidobacteria* and *OD1*.
(TIF)

Figure S4 **Composition of phyla per individual.** The fractions of the four most abundant phyla and changes over time are depicted. Individuals within a group are aligned based on abundance of *Firmicutes*. While there is an relatively stable composition per individual in the non-smoking (N1-5) and smoking (S1-5) control groups, obvious shifts in the subjects from the intervention group (I1-10) can be detected between the time points before (t1) and after (t2, t3) smoking cessation.
(TIF)

Figure S5 **Heat map with color-coded significance levels.** The results of paired Student's T-Test is depicted on the level of phylum (analogs to Main Figure 1b) including separate analysis for all genera constituting each phylum.
(TIF)

Figure S6 **Weighted PCA. Analog to Main** **Figure 2** **.** Findings are similar to the unweighted analysis. Again a clear separation of samples of the intervention group is obvious. Although there is some variation in the non-smoking Control group (N) as well, the difference is notably smaller (smoking control group  = S).
(TIF)

Figure S7 **Jackknife analysis.** In this statistical approach to test for reproducibility of results, resampling is done several times with a number of sequences (in general sample size of around 75% of the number of sequences in the sample with the lowest count is recommended (runs were performed with sample size 18000). To increase statistical power we set number of permutations to 1000. QIIME constructs a color coded bootstrapped tree (red  = 75–100% support, yellow  = 50–75%, green = 25–50% and blue for <25%; unweighted (A) and weighted (B) tree). The number besides the nodes indicates the percentage of how many times the node was reproduced during resampling. A perfectly high reproducibility can be seen.
(TIF)

Figure S8 **PD and PD-Endemism.** There are substantially higher values in the intervention group after smoking cessation. PD-Endemism refers to the amount of branch length that is uniquely represented by a certain environment while PD is indicative of the total phylogenetic branch length, that is spanned by all species included in the environment.
(TIF)

Figure S9 **Rarefaction curves (analog to main** **Figure 3b** **; curves express the accumulation of phylogenetic richness that would be obtained with continuous sampling effort and hence minimize potential differences that would be a result of the variable number of sequences obtained per sample).** In addition to the phylogeny-based PD whole tree (phylogenetic tree is needed for calculation of this α-diversity measure) (A), two further indices of α-diversity, chao1 (B) and observed species (C) are depicted. The latter two indices reveal a significantly increased α-diversity also 8 weeks after smoking cessation. For the control groups the three sampling time points were combined in a single curve, while for the intervention group separated curves for t1, t2 and t3 were depicted to visualize the increased microbial diversity (PD) in the samples 4 weeks after smoking cessation (I2) compared to I1 and I3.
(TIF)

Table S1 **Evolution of body weight and BMI in the 10 subjects undergoing smoking cessation.**
(TIF)

Table S2 **Overview of individual calorie intake in the 10 subjects undergoing smoking cessation.**
(TIF)

Table S3 **Paired T-Test (Comparison of total calories as well as fraction of carbohydrates, proteins, fat fibers and alcohol between t1, t2 and t3).** With the exception of calories from alcohol between t2 and t3 no significant changes are detected.
(TIF)

Table S4 **Summary of additive main effects and multiplicative interaction model (AMMI) results.** Table **A** shows the results of the analysis of variance (ANOVA) with a two-way data matrix (df  =  degrees of freedom, SS  =  sum of squares and MS  =  mean square). Table **B** shows the interaction sums of squares (SS), giving an estimate of the contribution of idiosyncratic noise to the observed pattern, which in this case is regarded as meaningful. However, the value for the interaction pattern is clearly positive (a negative value would occur if the estimated interaction noise is more meaningful than the interaction total). Table **C** shows the per cent of variation from each source in AMMI (while main effects variation reflects variation from T-RFs and Environments, replicated data interaction effects reflects interaction pattern and interaction noise; variation due to Interaction Effects reflect dissimilarity of the investigated microbial communities). Table **D** shows how much of the predicted interaction signal variation is captured in the first IPCAs (in the table the first four IPCAs are depicted; here complete capture is already achieved with 3 axes.
(TIF)

Text S1 (DOCX)
