# Supplementary figures and images for: Smoking Cessation Induces Profound Changes in the Composition of the Intestinal Microbiota in Humans
Source: PLoS One. 2013 Mar 14;8(3):e59260. doi: 10.1371/journal.pone.0059260 (PMC3597605; doi:10.1371/journal.pone.0059260)

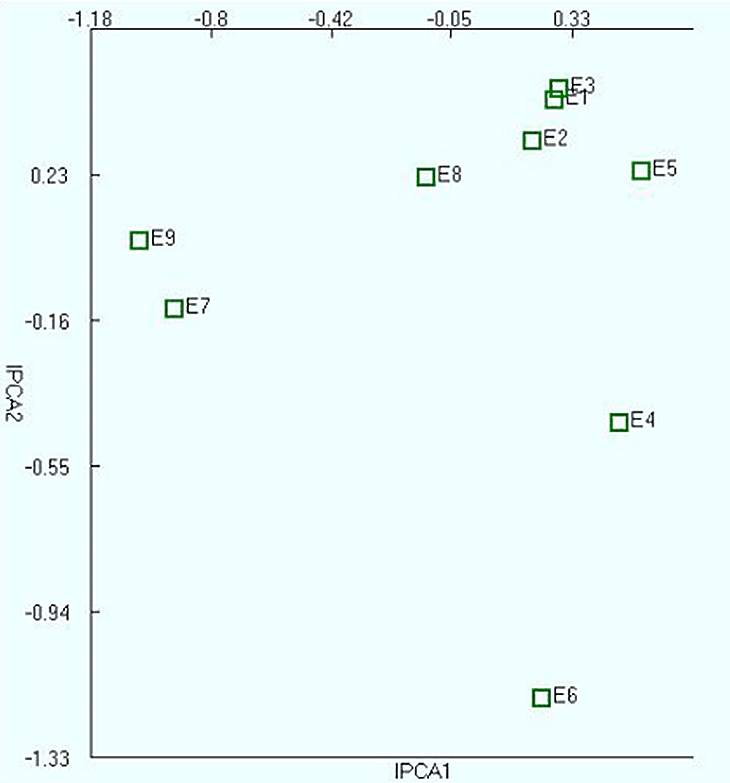

Supplement: Figure S2 — PCA with 9 environments. Separation of the samples from the intervention group (E7 = I, t1; E8 = I, t2; E9 = I, t3) alongside the first principal component axis (IPCA1) reflecting the largest difference, 58.5% (as outlined in Table D, Table S4.). There is a close association of samples from the non-smoking control group (E1 = N, t1; E2 = N, t2; E3 = N, t3). A wider separation is to be seen in the smoking control group (E4 = S, t1; E5 = S, t2; E6 = S, t3), however almost exclusively alongside the second principal component axis (IPCA2), reflecting a substantially smaller difference (37.7%). (TIF) [file pone.0059260.s002.tif]

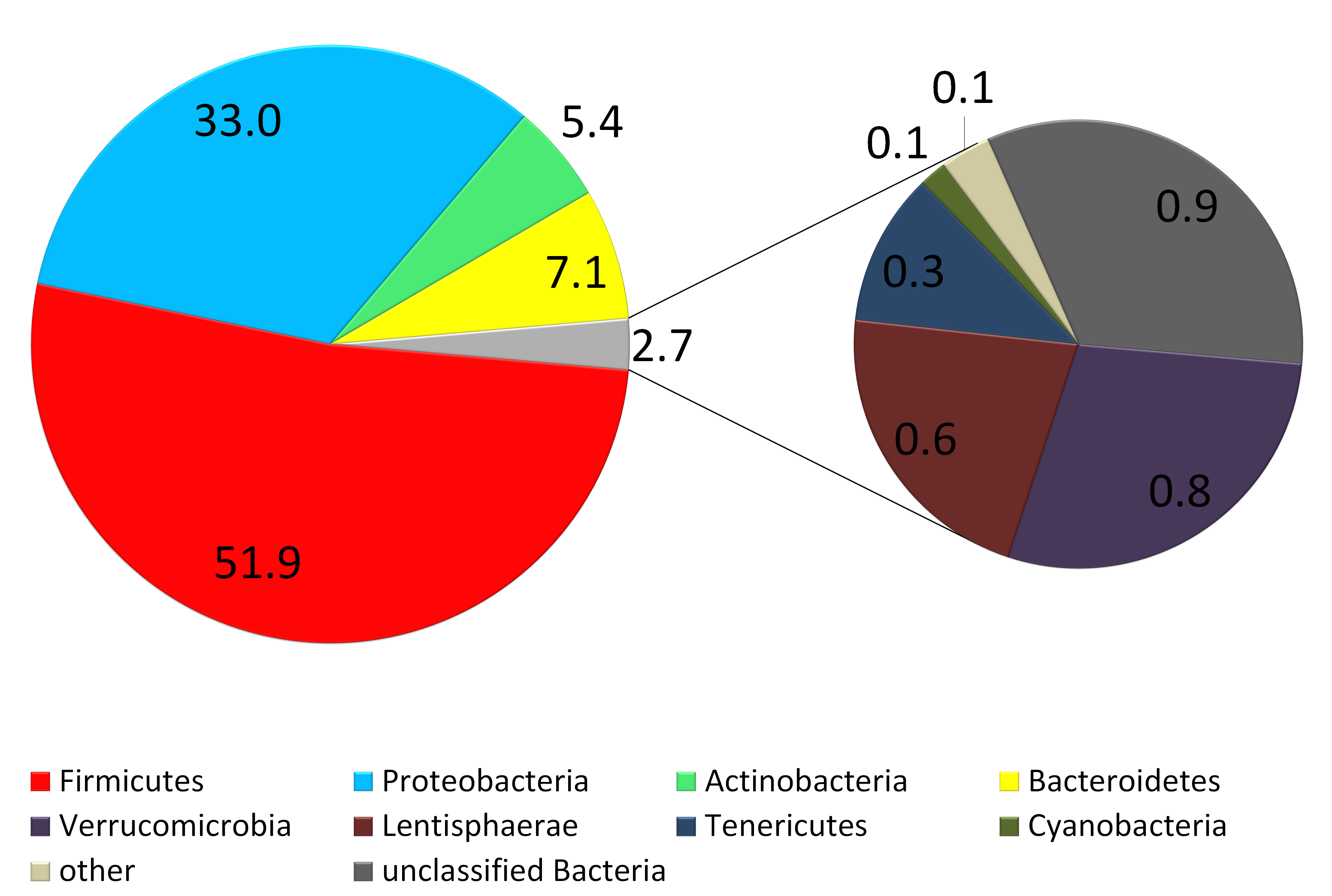

Supplement: Figure S3 — Distribution of phyla in all samples in percentages. The fraction other (0.1% of all sequences) in the right pie chart consists of Synergistetes, Fusobacteria, Deinococcus-Thermus, TM7, Acidobacteria and OD1. (TIF) [file pone.0059260.s003.tif]

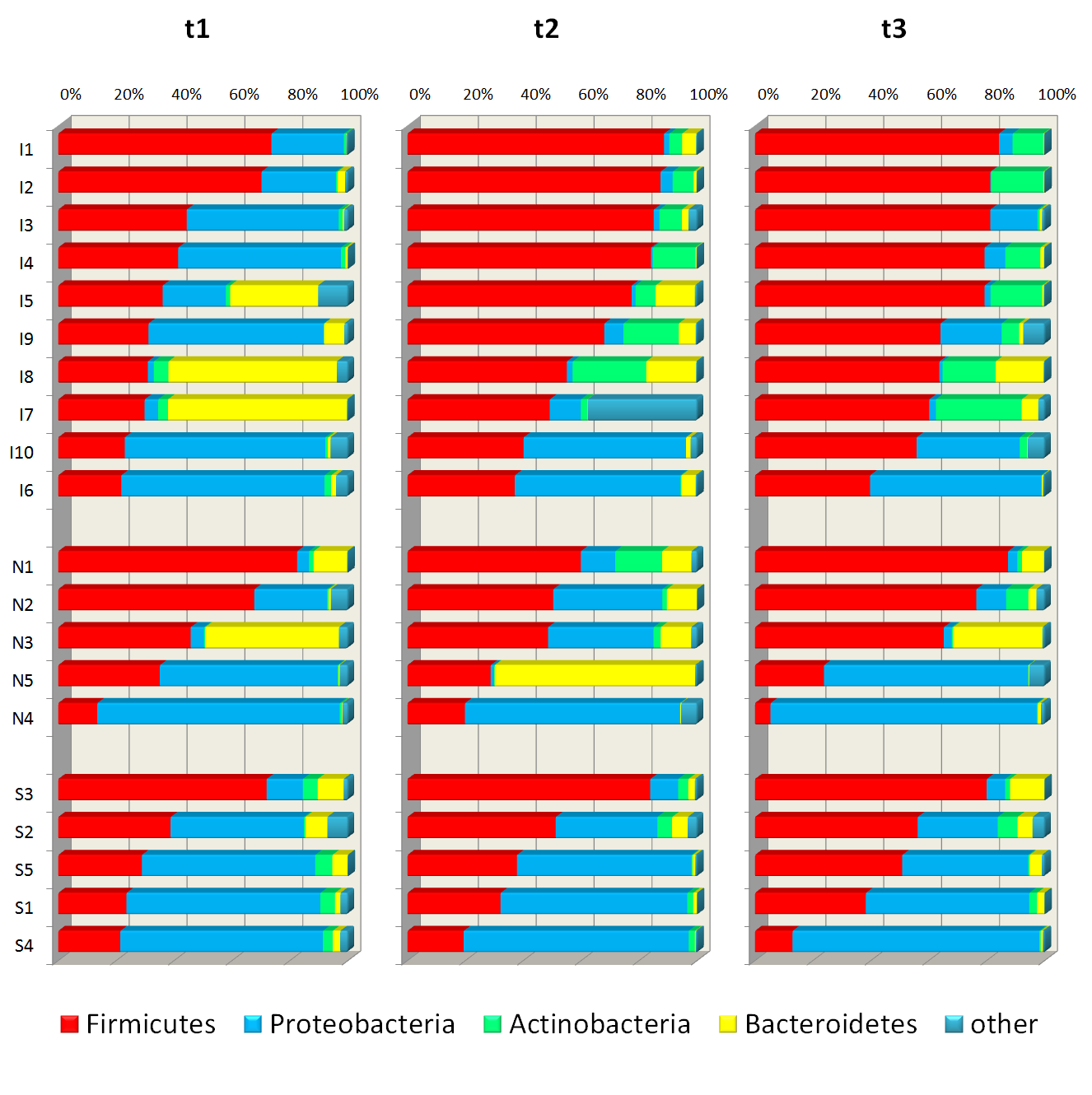

Supplement: Figure S4 — Composition of phyla per individual. The fractions of the four most abundant phyla and changes over time are depicted. Individuals within a group are aligned based on abundance of Firmicutes. While there is an relatively stable composition per individual in the non-smoking (N1-5) and smoking (S1-5) control groups, obvious shifts in the subjects from the intervention group (I1-10) can be detected between the time points before (t1) and after (t2, t3) smoking cessation. (TIF) [file pone.0059260.s004.tif]

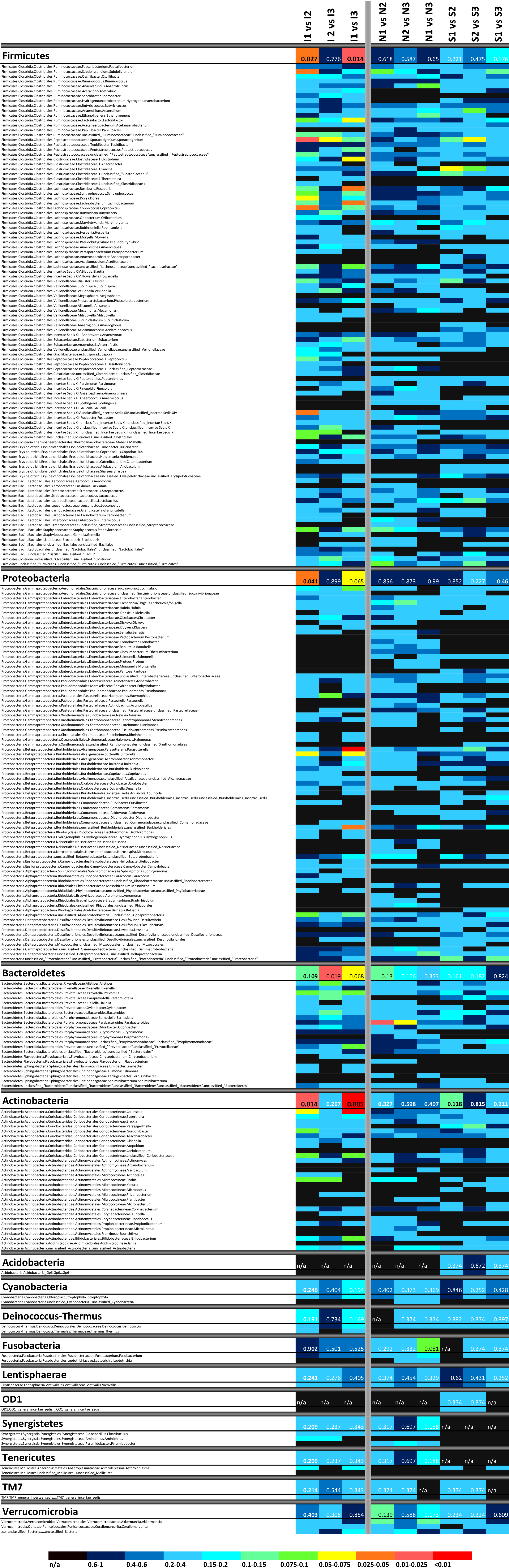

Supplement: Figure S5 — Heat map with color-coded significance levels. The results of paired Student's T-Test is depicted on the level of phylum (analogs to Main Figure 1b) including separate analysis for all genera constituting each phylum. (TIF) [file pone.0059260.s005.tif]

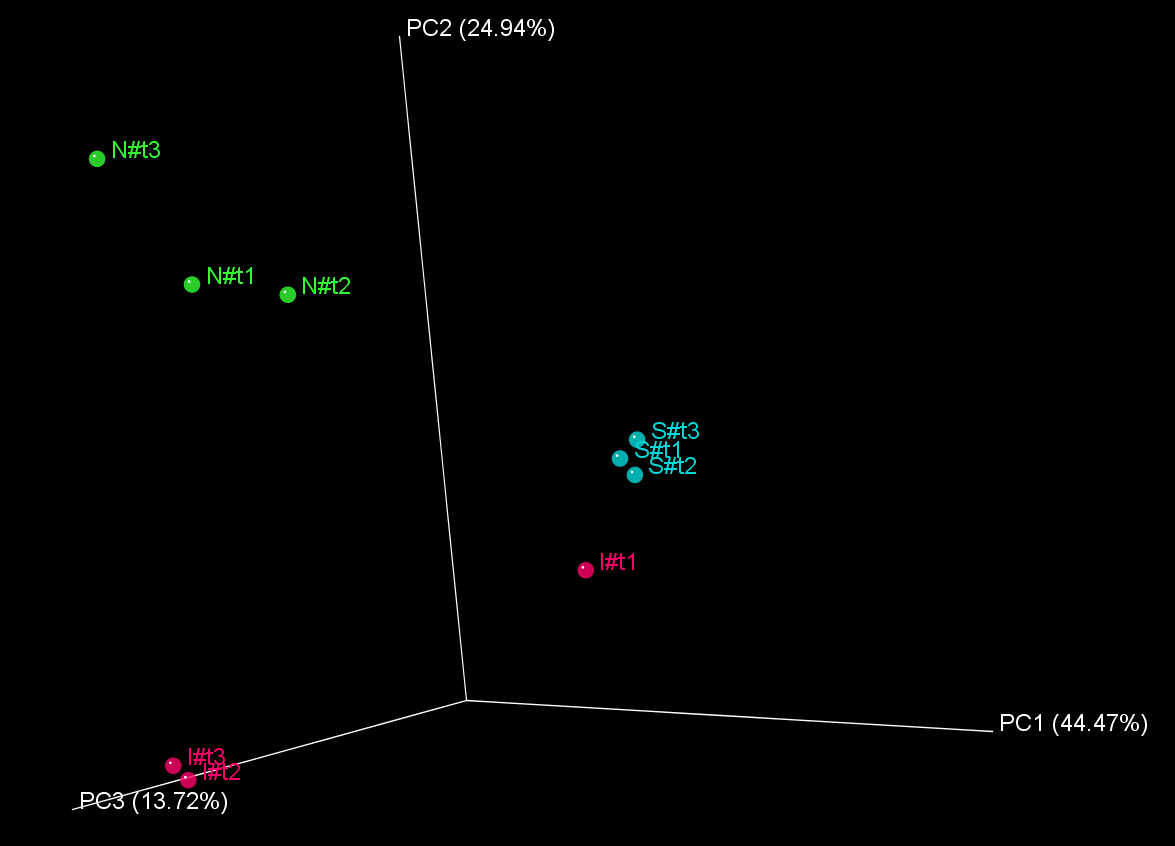

Supplement: Figure S6 — Weighted PCA. Analog to Main Figure 2 . Findings are similar to the unweighted analysis. Again a clear separation of samples of the intervention group is obvious. Although there is some variation in the non-smoking Control group (N) as well, the difference is notably smaller (smoking control group = S). (TIF) [file pone.0059260.s006.tif]

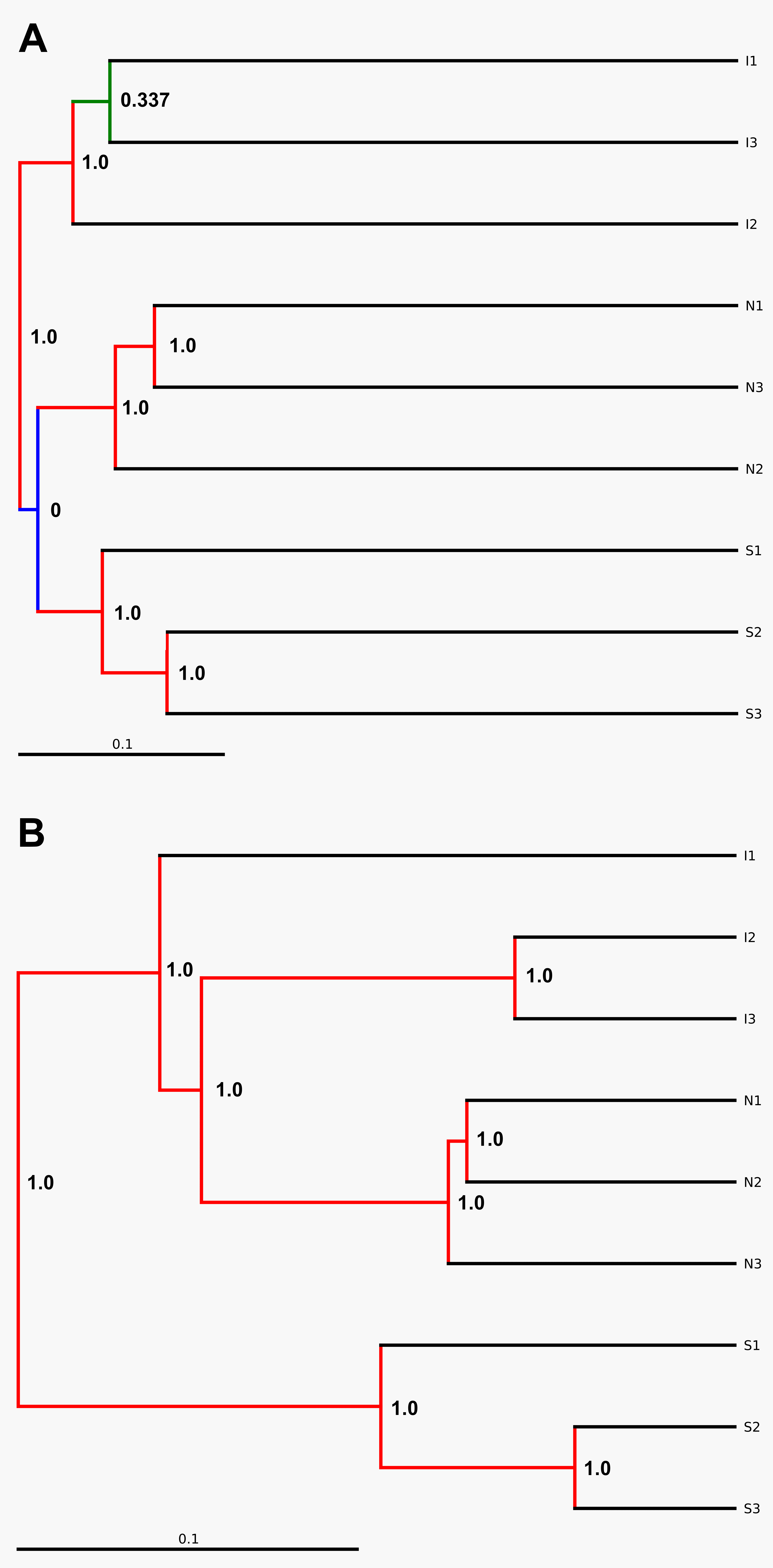

Supplement: Figure S7 — Jackknife analysis. In this statistical approach to test for reproducibility of results, resampling is done several times with a number of sequences (in general sample size of around 75% of the number of sequences in the sample with the lowest count is recommended (runs were performed with sample size 18000). To increase statistical power we set number of permutations to 1000. QIIME constructs a color coded bootstrapped tree (red = 75–100% support, yellow = 50–75%, green = 25–50% and blue for <25%; unweighted (A) and weighted (B) tree). The number besides the nodes indicates the percentage of how many times the node was reproduced during resampling. A perfectly high reproducibility can be seen. (TIF) [file pone.0059260.s007.tif]

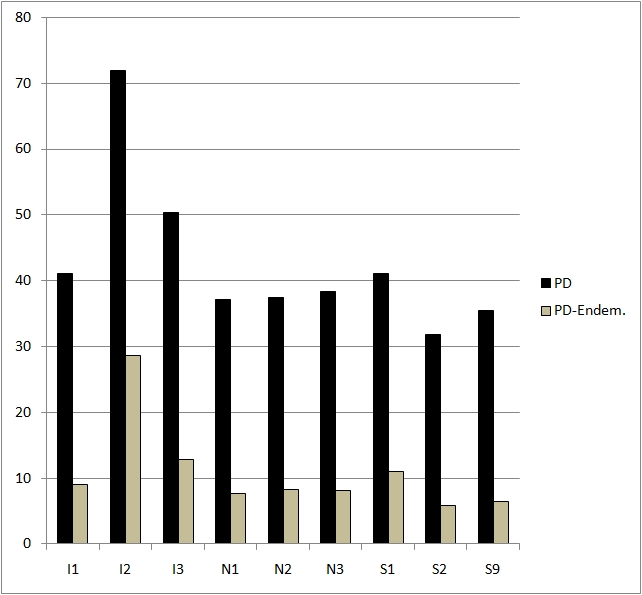

Supplement: Figure S8 — PD and PD-Endemism. There are substantially higher values in the intervention group after smoking cessation. PD-Endemism refers to the amount of branch length that is uniquely represented by a certain environment while PD is indicative of the total phylogenetic branch length, that is spanned by all species included in the environment. (TIF) [file pone.0059260.s008.tif]

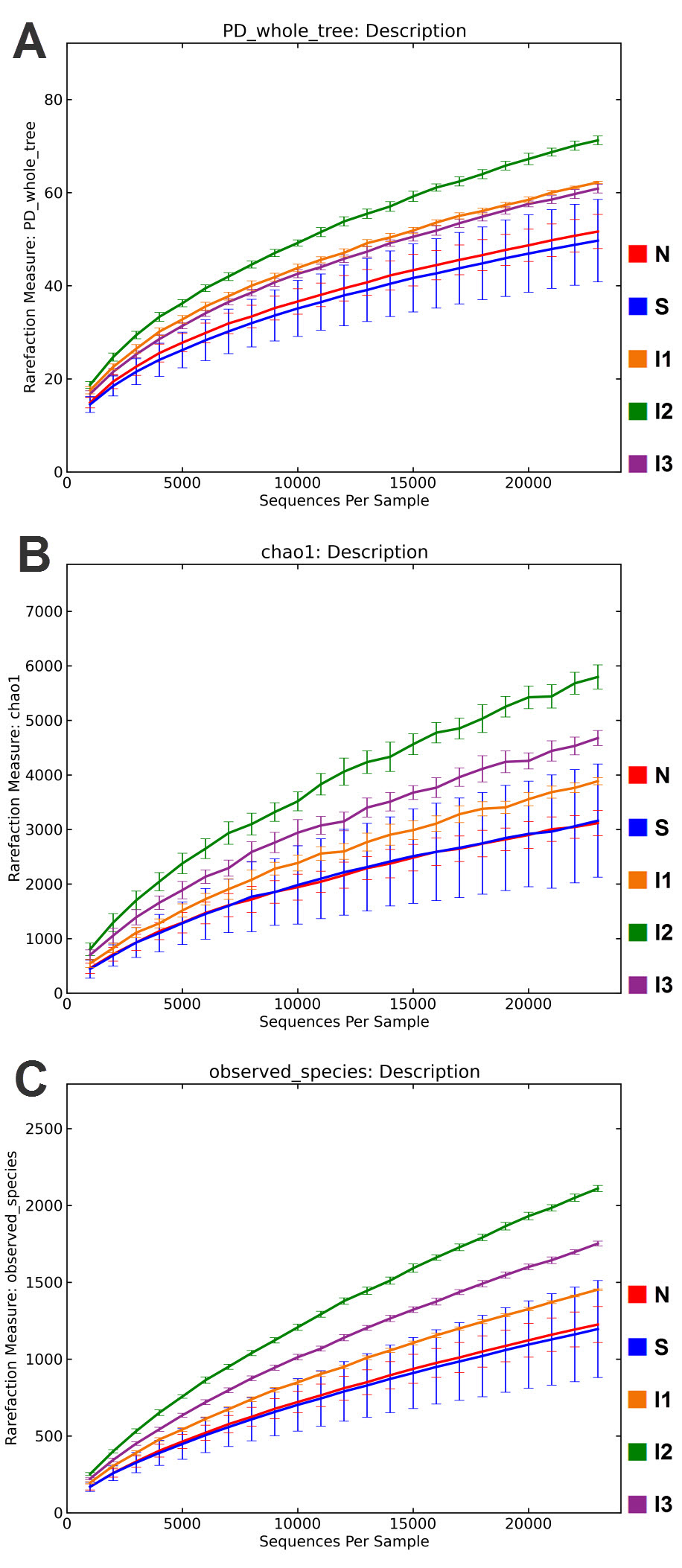

Supplement: Figure S9 — Rarefaction curves (analog to main Figure 3b ; curves express the accumulation of phylogenetic richness that would be obtained with continuous sampling effort and hence minimize potential differences that would be a result of the variable number of sequences obtained per sample). In addition to the phylogeny-based PD whole tree (phylogenetic tree is needed for calculation of this α-diversity measure) (A), two further indices of α-diversity, chao1 (B) and observed species (C) are depicted. The latter two indices reveal a significantly increased α-diversity also 8 weeks after smoking cessation. For the control groups the three sampling time points were combined in a single curve, while for the intervention group separated curves for t1, t2 and t3 were depicted to visualize the increased microbial diversity (PD) in the samples 4 weeks after smoking cessation (I2) compared to I1 and I3. (TIF) [file pone.0059260.s009.tif]

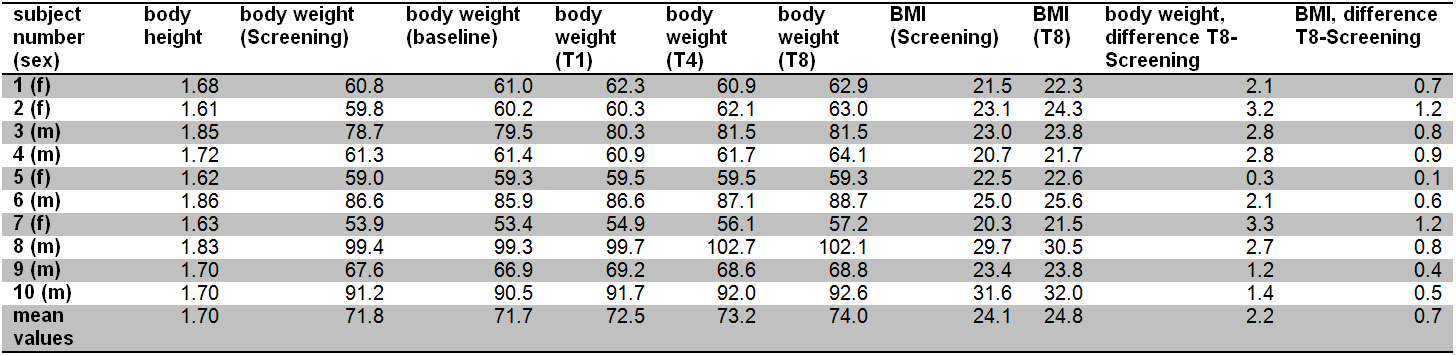

Supplement: Table S1 — Evolution of body weight and BMI in the 10 subjects undergoing smoking cessation. (TIF) [file pone.0059260.s010.tif]

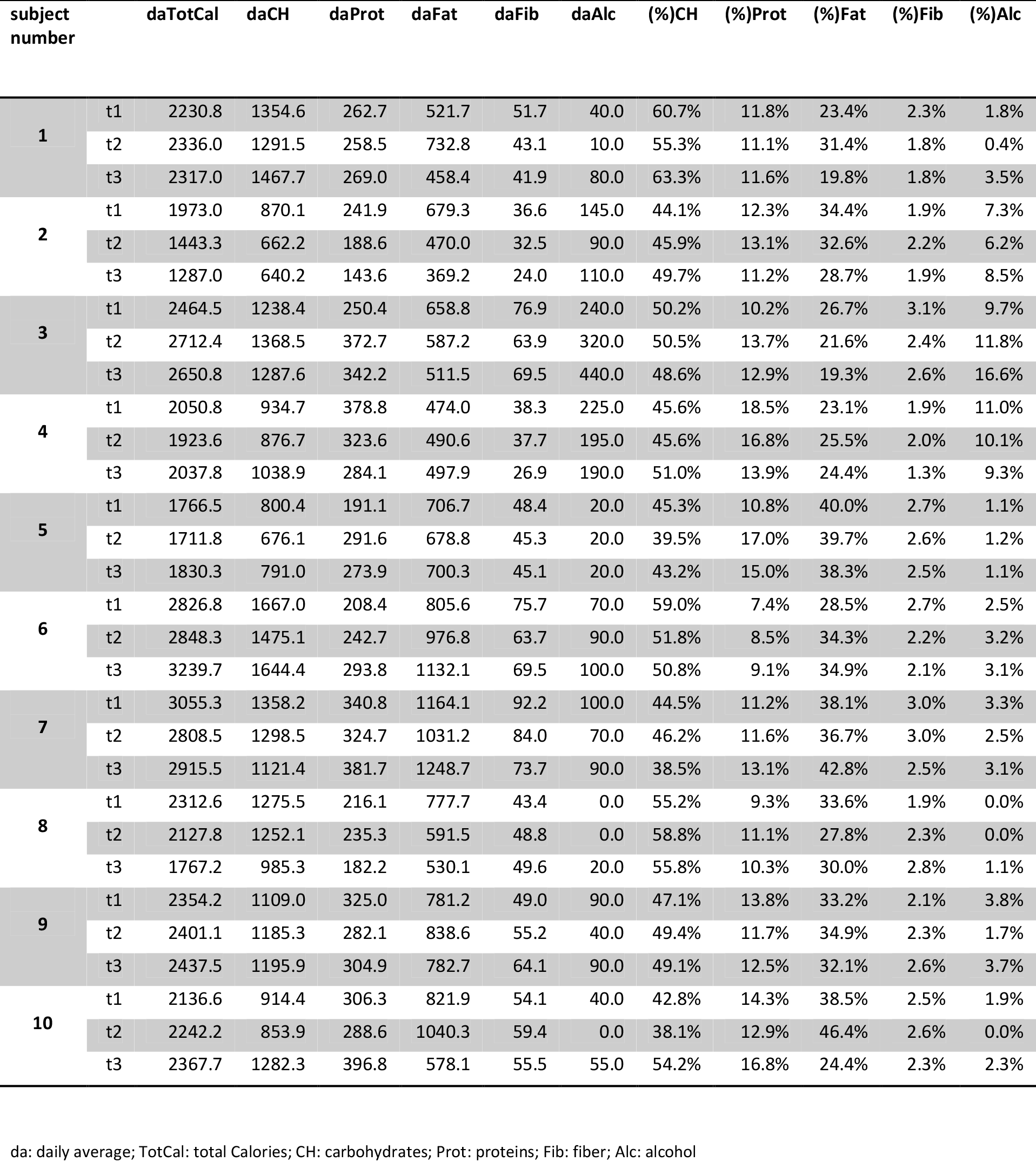

Supplement: Table S2 — Overview of individual calorie intake in the 10 subjects undergoing smoking cessation. (TIF) [file pone.0059260.s011.tif]

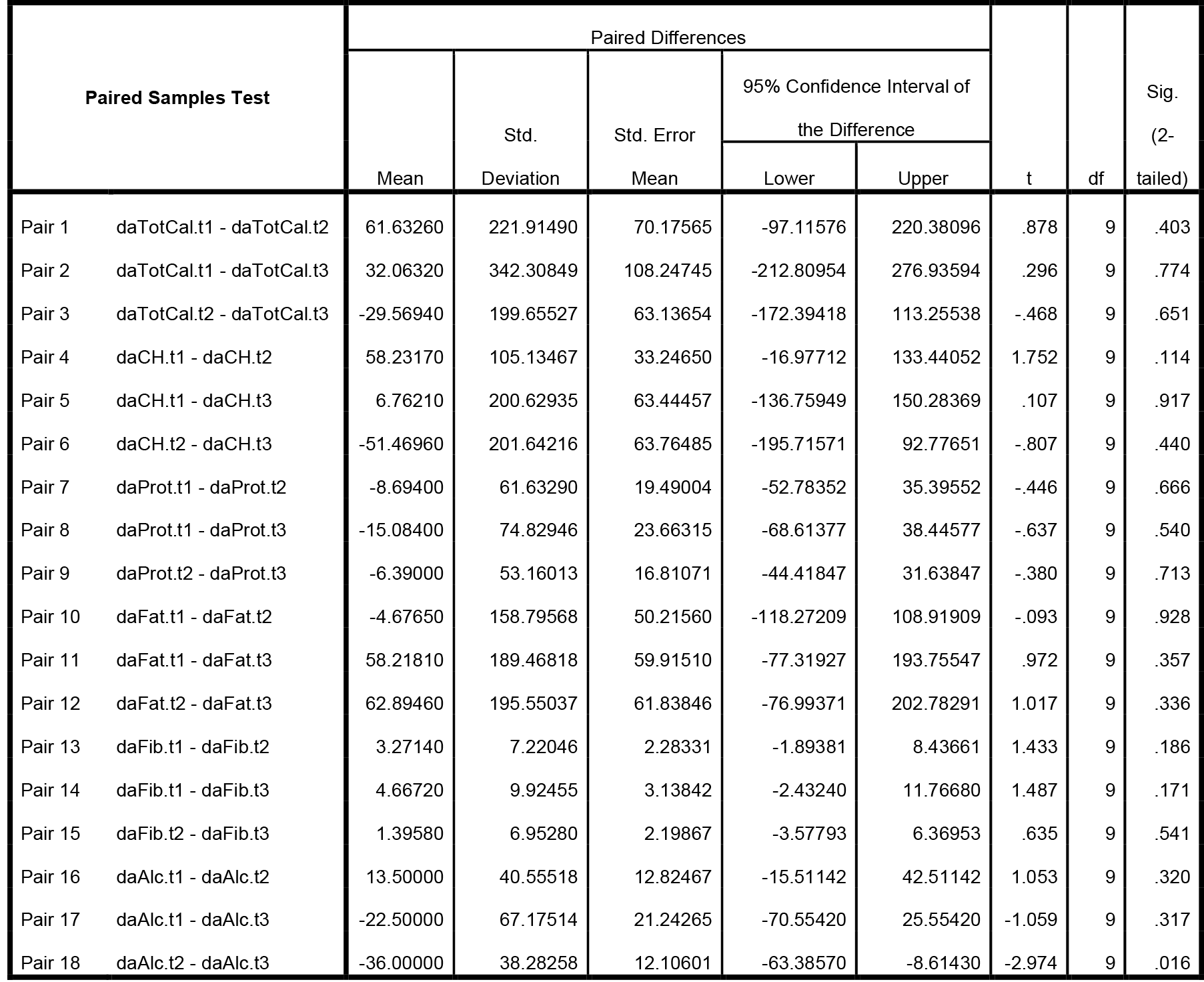

Supplement: Table S3 — Paired T-Test (Comparison of total calories as well as fraction of carbohydrates, proteins, fat fibers and alcohol between t1, t2 and t3). With the exception of calories from alcohol between t2 and t3 no significant changes are detected. (TIF) [file pone.0059260.s012.tif]

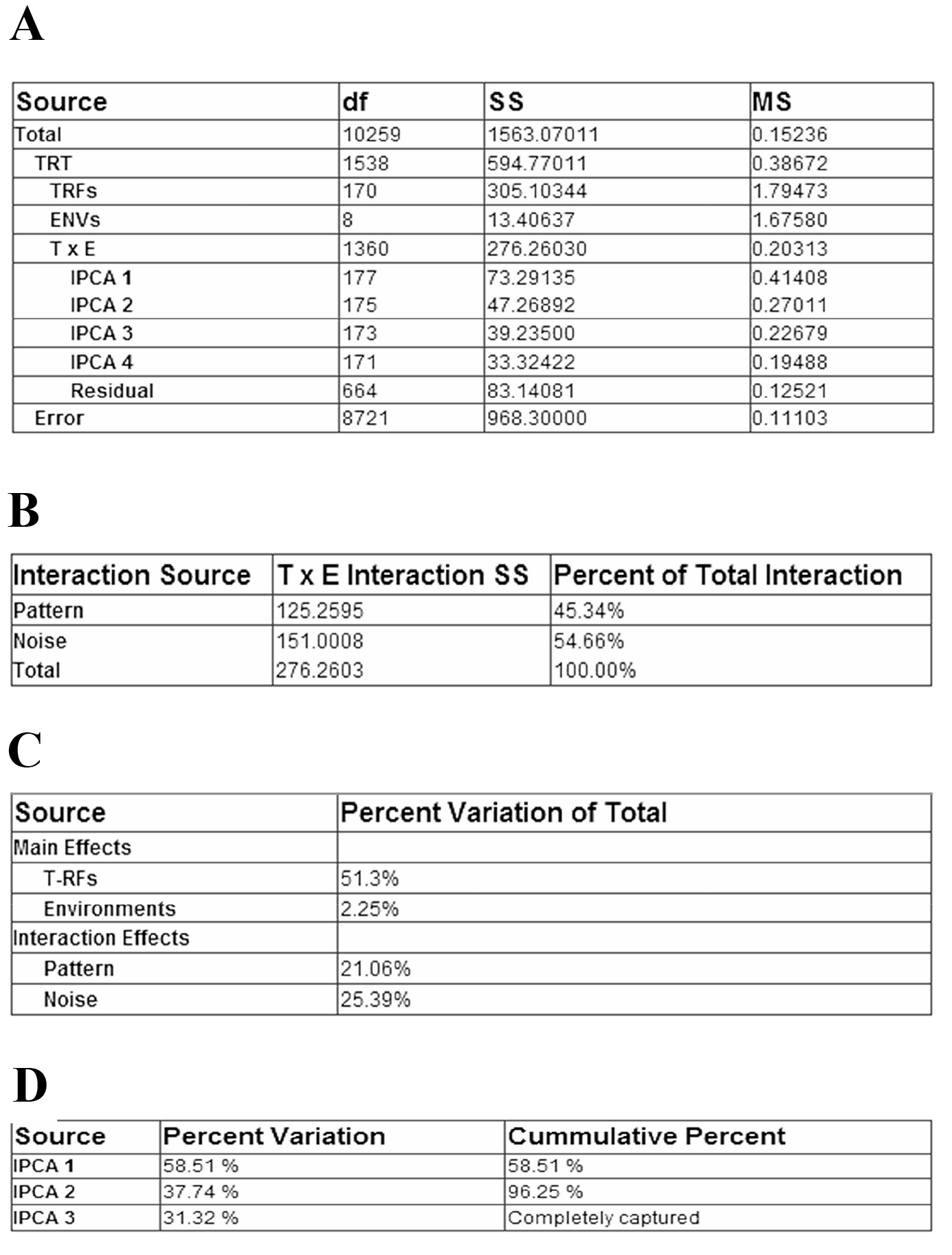

Supplement: Table S4 — Summary of additive main effects and multiplicative interaction model (AMMI) results. Table A shows the results of the analysis of variance (ANOVA) with a two-way data matrix (df = degrees of freedom, SS = sum of squares and MS = mean square). Table B shows the interaction sums of squares (SS), giving an estimate of the contribution of idiosyncratic noise to the observed pattern, which in this case is regarded as meaningful. However, the value for the interaction pattern is clearly positive (a negative value would occur if the estimated interaction noise is more meaningful than the interaction total). Table C shows the per cent of variation from each source in AMMI (while main effects variation reflects variation from T-RFs and Environments, replicated data interaction effects reflects interaction pattern and interaction noise; variation due to Interaction Effects reflect dissimilarity of the investigated microbial communities). Table D shows how much of the predicted interaction signal variation is captured in the first IPCAs (in the table the first four IPCAs are depicted; here complete capture is already achieved with 3 axes. (TIF) [file pone.0059260.s013.tif]
