# Supplementary material for: Smoking Cessation Induces Profound Changes in the Composition of the Intestinal Microbiota in Humans
Source: PLoS One. 2013 Mar 14;8(3):e59260. doi: 10.1371/journal.pone.0059260 (PMC3597605; doi:10.1371/journal.pone.0059260)
Supplement: Text S1 — (DOCX) [file pone.0059260.s014.docx]

**Supporting Information Text**

SI Materials and Methods

Study design

**Smoking cessation.** The average continuous low abstinence rates of around 1.4% after 1 year for usual care can be increased to 6.0% with intensive counseling and to 12.3% with supportive pharmacotherapy [1]. However, as an influence of any additional pharmacotherapy to support successful smoking cessation on the intestinal microbiota cannot be ruled out, neither additional pharmacological support was allowed, nor the use of antibiotics in the previous 3 weeks before study inclusion.

**Food frequency protocol.** Study subjects had to complete a daily food-frequency protocol three times over a period of 7 consecutive days (one week before t1, t2, and t3; validated Food-Frequency Protocol, Prof. V. Pudel, Goettingen, Germany). Each daily food diary (to be answered over a period of 7 consecutive days; one week before t1, t2 and t3) contained a list of conventional meals and drinks to be checked as well as additional space to mention specific food not included in the list. Thus food pattern could be assessed on a qualitative and semi-quantitative manner.

**Stool sample collection.** Study subjects received detailed written and/or oral instructions on how to collect stool samples together with plastic tubes and a receiving bowl by conventional mail prior to the screening visit. Stool samples were collected in conventional fecal samples storage tubes including a shovel attached to the cap. On every study visit (S, T0, T1, T4 and T8) study subjects were to return 2 storage tubes. One tube was left empty (native stool) the other contained 10ml PBS/Ethanol (50% v/v of Phosphate Buffered Saline (PBS) and Ethanol 96%), the latter leading to an immediate denaturation of bacterial cell wall. For further analyses only the samples in PBS/Ethanol were used. Subjects were instructed to collect a quantity of stool roughly corresponding to the size of a cherry per tube, and to verify complete dipping of the stool sample into the PBS/ethanol mixture containing tube. The stool had always to be collected from a bowel movement either on the day of a study visit or one day before at maximum, and was to be stored immediately after collection in the freezer (-20°C, native stool) and refrigerator (4-6°C, stool sample in PBS/ethanol) respectively. Although it is debatable to what extent stool samples are representative of the intestinal microbiota [2], they are used in most human studies investigating human intestinal microbiota and potential modulating effectors as they are easily and repetitively attainable. Stool samples have been shown to be representative of inter-individual differences in composition of microbiota [3].

T-RFLP and DNA extraction

The fixed fecal samples were thoroughly homogenized. An aliquot of 2 ml of the homogenized samples was centrifuged (12’000 x g, 1 min), washed twice with phosphate-buffered-saline (PBS) and finally re-suspended in 0.5 ml of PBS. Total nucleic acids were extracted as described by Schuppler et al. [4].
PCR was performed using two universal bacteria primers (TPU1 FAM: 5’-AGA GTT TGA TCM TGG CTC AG-3’, a fluorescent primer which produces 5’ terminally labeled PCR products, only the terminal fragments carrying the fluorescent label can be detected during capillary electrophoresis in the genetic analyzer; and RTU4: 5’-ACC AGG GTA TCT AAT CCT G-3’) binding to highly conserved regions of 16S rRNA genes. With agarose gel electrophoresis the success of the amplification reaction was verified. After a mung bean nuclease digest and purification reaction to remove single stranded DNA fragments a digestion with 2 restriction enzymes (HhaI and MspI) was performed. An internal length standard (Map Marker) and formamid were administered in the digestion reaction before loading samples in the genetic analyzer. GenScan® analysis software was used to analyze the output. T-RFLP Operation Results Analysis Software Tool (TORAST) and T-RFLP Analyses Program (TAP) were used for identification of potential corresponding microorganisms to the obtained T-RF lengths (virtual digest was performed considering the utilized primers and restriction enzymes; imprecision of plus/minus one base pair was tolerated). Corresponding phylum, class, order, family and genus to a respective terminal restriction fragment length were determined by means of the taxonomy browser on the National Center for Biotechnology Information (NCBI) site.

Pyrosequencing

Genomic DNA from fecal pellets was extracted by MO BIO PowerSoil® DNA Isolation kit with some modification. Solution C1 and 20µl of Proteinase K was added to feces and incubated for 2 hours at 50°C to enhance the lysis. The rest of the protocol was followed according to the manufacturer’s recommendations. Extracted DNA was quantified by using the Quant-iT™ PicoGreen® dsDNA Assay Kit (Invitrogen®). We amplified variable region V1-V2 of the 16S rRNA gene using forward primer (5´-**CTATGCGCCTTGCCAGCCCGC**TCAGTCAGAGTTTGATCCTGGCTCAG-3´; sequence in bold face is 454 Life Sciences® primer B, the underlined region represents the sequence of the universal bacterial primer 27F (*E. coli* 16S rRNA gene position 8 – 27); two-base linker sequence (‘TC’) and four base key were added in accordance with the Roche® recommendations. The reverse primer (5´-**CGTATCGCCTCCCTCGCGCCA**TCAGXXXXXXXXXXCATGCTGCCTCCCGTAGGAGT-3´) contained the 454 Life Sciences® primer A sequence (bold), and unique 10 base multiplex identifier (MIDs designated as XXXXXXXXXX) to tag each Polymerase Chain Reaction (PCR) product, the underlined universal bacterial primer 338R (*E. coli* 16S rRNA gene position 338 – 355) and a ‘CA’ linker sequence inserted between the MIDs and the rRNA gene primer. A four base key was added in front of the MIDs in the reverse primer. Replicate PCRs were performed for each sample and negative controls. Each reaction consisted of 1ul (10pmol/ul) of each primer, 40 ng of DNA, 1 ul of dNTPs mix (10mM stock), 1.0 units of Phusion® Hot Start II High-Fidelity DNA Polymerase and 10ul of 5x Phusion® HF buffer. Final volume of reaction was adjusted to 50 ul by adding PCR grade water. Amplification was performed using an initial denaturation of 3 min at 98°C followed by 30 cycles, denaturation at 98°C for 10 seconds, annealing at 55°C for 30 seconds and elongation at 72°C for 30 seconds. Final extension was at 72°C for 10 minutes. Amplified product was run on agarose gel, specific band was excised and amplicons were purified by Qiagen™ gel purification kit. Concentration of eluted and purified amplicons was measured by Quant-iT™ PicoGreen® dsDNA Assay Kit (Invitrogen®). Equal amount of PCR products were mixed in a single tube and sequenced using Roche® 454 titanium chemistry. Replicate PCRs were performed for each sample as well as negative controls (water).

Phylogenetic analysis of sequence data - Selection of sequences, tree building

After the sequencing procedure all sequence reads were screened and filtered for quality and length using perl script [5]. Thereafter, sequences were trimmed and the specific MIDs (barcode) allowed allocation of sequences to the originating sample. The following quality criteria were to be fulfilled, otherwise sequences were excluded:

- having perfect match with MID sequences or specific primers
- more than 200 base long reads
- quality score equal or more than 20
- not having any ambiguous base
- maximal 6 homopolymers per sequence

Chimeric sequences were removed with MOTHUR chimera slayer [6]. Further quality control was achieved with comparison to the SILVA database [7]. In total we generated 335’902 sequences fulfilling quality criteria. These could be assigned to the 66 samples (10 subjects in the intervention group, 5 subjects in each smoking and non-smoking control group at 3 time points, 3 incomplete subjects from the intervention group only represented by samples t1 and t2).

Statistics

From the cd-hit-output of clusters which were grouped by cluster size and sequence length the longest sequence was chosen as a representative of the cluster. Alignment of sequences was done with NAST, a multiple sequence alignment server for comparative analysis of 16S rRNA genes from the Greengenes server [8] (minimum length 150). The output from the alignment procedure in the greengenes 7,682-character format was inserted subsequently in masked columns, a tool for masking hyper-variable regions from the aligned sequences also from the greengenes server. Phylogenetic tree building was done with FastTree 2 [9], a tree constructing software, also suitable for larger trees providing phylogenetic trees in the Newick format.

**SI Results**

Pyrosequencing

Considering the attributed taxonomy to each sequence we found a predominance of four phyla. Together, sequences belonging to one of these major four phyla accounted for 97.3% of all sequences.

As outlined in Figure S3 all other sequences were assigned to the following phyla: *Verrucomicrobia, Lentisphaerae, Tenericutes, Cyanobacteria, Synergistetes, Fusobacteria, Deinococcus-Thermus, TM7, Acidobacteria* and *OD1* constituted only a small fraction of the total intestinal microbiota. Only a small amount of sequences (0.9%) could not be classified. An overview of the changes of microbial composition in each individual is given in Figure S4.

The result of paired Student´s t-test is shown on the phylum and genus level in a color-coded heat map (Figure S5). Significance levels are shown in different colors (shades of red, significant shifts in bacteria composition; shades of yellow, green and blue, non-significant shifts) and are indicated by the exact significance values within the colored squares of the graph. The major changes in the microbiota in the intervention group are observed between the time points before (t1) and after (t2, t3) smoking cessation. In contrast no significant changes are detected in the control groups and - with the exception of *Bacteroidetes* – after smoking cessation between t2 and t3 in the intervention group.

**Normalized set of sequences with 1000 OTUs per sample**

The mean number of sequences or OTUs derived by pyrosequencing per sample was 5321 (std. error 471). The reason for this heterogeneity is not clear. As the pyrosequencing was performed on a single plate (one single run) harboring all stool samples, the conditions for all samples from polymerization to light detection were identical, i.e. the probability of detection of a certain fragment of 16S rRNA gene by pyrosequencing can be assumed to be similar in all study samples. Most likely the differences in sequences obtained per sample may be reflective of other factors, such as differences in bacterial density per individual stool sample or simply by chance. We thus decided to use all obtained sequences without any normalization step for the number of sequences per sample for all consecutive analyses. For the OTU-based analyses of sequence abundance the number of sequences is not likely to affect the final result anyway, as percentages of the total number of sequences per sample were taken into account.

Nevertheless, to rule out a potential skewing of data due to differences in sequence numbers per sample, we independently re-performed a variety of the analyses using a normalized set of sequences with 1000 OTUs per sample. Therefore 1000 OTUs from each of the 60 samples were randomly picked by QIME with a multiple rarefaction script. Only in 9 of the 60 samples, the OTU count was below 1000; thus in these samples, the full number of OTUs were used. According to this 10 different rarefied OTU tables were generated, which were combined to a single OTU table (with an equal number of OTUs derived per sample, with the exception of the samples with lower than 1000 OTUs). The following results were obtained after this standardization process (*Figures not shown, may be provided subsequently*):

Similar to the main results including all picked OTUs, UniFrac distance revealed to be substantially higher with the normalized OTUs per sample analyses in the intervention group before and after smoking cessation. The results of the rarefaction plots and increases in alpha diversity were confirmed. Yet, the increase appeared to be even more pronounced and sustained over the whole observation period of eight weeks. However, as in the main analyses, again a decrease in diversity values (both phylogeny based and non-phylogeny based) 8 weeks after smoking cessation compared to 4 weeks after smoking cessation was noted. We furthermore performed bootstrapping analyses of β-diversity with the normalized set of sequences. Again PCA indicated the most pronounced separation of samples in the intervention group between t1 and t2. Likewise, separation of bacterial lineages again was observed closer to the root of the bootstrapped tree in both, weighted and unweighted analyses. The nodes generated by randomly picking 30000 OTUs from each sample (around 75% of the number of sequences in the sample with the lowest count) with 1000 permutations revealed a very high (unweighted) or perfect (weighted) reproducibility.

**SI Discussion**

In recent years a magnitude of studies investigated the composition of the commensal microbiota in IBD patients compared to healthy controls or patients with other diseases. These studies showed several interesting and independently reproduced findings, such as a reduced microbial diversity in patients with IBD [10–13]. Moreover, distinctive characteristics of microbial composition in patients with IBD compared to healthy controls have been described, albeit sometimes with conflicting results. These include alterations on the level of phylum, such as a reduction of *Firmicutes* and *Bacteroidetes* and concomitant increase in the fraction of *Proteobacteria* and *Actinobacteria* [14–16]. Also on the level of species [17] alterations have been described, such as a combined decrease of *Dialister invisus* (an hitherto uncharacterized member of the *Clostridiacae family*), *Faecalibacterium prausnitzii*, *Biﬁdobacterium adolescentis* and an increase in *Ruminococcus gnavus* to be indicative of dysbiosis associated with CD [18].

However, it has to be bared in mind that even in large-scale clone library analysis comparing the microbiota of IBD patients and controls no specific pathogens in the sense of a causative bacterial agent could be identified [19,14,20]. Moreover, the armada of comparative sequence based studies investigating microbial composition give associative and descriptive information, as does our study, but not mechanistic or causative evidence [19].

According to this and in view of the relatively small number of sequences within lower bacterial divisions, we cannot draw any robust conclusions, whether the observed microbial shifts after smoking cessation are to be as considered as beneficial with regard to certain bacterial species, such as for instance the *Bifidobacteria*. The latter has been attributed to beneficial effects in both, the metabolic syndrome [21] and IBD [18,17,22–24].

**SI References**

1. Hoogendoorn M, Feenstra TL, Hoogenveen RT, Rutten-van Molken MPMH (2010) Long-term effectiveness and cost-effectiveness of smoking cessation interventions in patients with COPD. Thorax 65 (8): 711–718.

2. Savage DC (1977) Microbial ecology of the gastrointestinal tract. Annu. Rev. Microbiol. 31: 107–133. Available: doi:10.1146/annurev.mi.31.100177.000543.

3. Eckburg PB, Bik EM, Bernstein CN, Purdom E, Dethlefsen L et al. (2005) Diversity of the human intestinal microbial flora. Science 308 (5728): 1635–1638. Available: doi:10.1126/science.1110591.

4. Schuppler M, Mertens F, Schon G, Gobel UB (1995) Molecular characterization of nocardioform actinomycetes in activated sludge by 16S rRNA analysis. Microbiology 141 (2): 513–521. Available: http://mic.sgmjournals.org/cgi/content/abstract/141/2/513.

5. Giongo AFW (2010) PANGEA: pipeline for analysis of next generation amplicons. The ISME journal 4 (7): 852–861. Available: http://sfx.metabib.ch:9003/sfx_uzh?sid=Entrez%3APubMed&id=pmid%3A20182525.

6. Schloss P (2005) Introducing DOTUR, a computer program for defining operational taxonomic units and estimating species richness. Applied and environmental microbiology 71 (3): 1501–1506. Available: http://sfx.metabib.ch:9003/sfx_uzh?sid=Entrez%3APubMed&id=pmid%3A15746353.

7. Pruesse E, Quast C, Knittel K, Fuchs BM, Ludwig W et al. (2007) SILVA: a comprehensive online resource for quality checked and aligned ribosomal RNA sequence data compatible with ARB. Nucleic Acids Research 35 (21): 7188–7196.

8. DeSantis TZ, Hugenholtz P, Keller K, Brodie EL, Larsen N et al. (2006) NAST: a multiple sequence alignment server for comparative analysis of 16S rRNA genes. Nucleic Acids Research 34 (Web Server): W394.

9. Price MN, Dehal PS, Arkin AP (2009) FastTree: Computing Large Minimum Evolution Trees with Profiles instead of a Distance Matrix. Molecular Biology and Evolution 26 (7): 1641–1650.

10. Manichanh C (2006) Reduced diversity of faecal microbiota in Crohn's disease revealed by a metagenomic approach. Gut 55 (2): 205–211.

11. Sokol H, Lay C, Seksik P, Tannock GW (2008) Analysis of bacterial bowel communities of IBD patients: what has it revealed. Inflamm. Bowel Dis. 14 (6): 858–867. Available: doi:10.1002/ibd.20392.

12. Martinez C, Antolin M, Santos J, Torrejon A, Casellas F et al. (2008) Unstable Composition of the Fecal Microbiota in Ulcerative Colitis During Clinical Remission. Am J Gastroenterol 103 (3): 643–648. Available: http://dx.doi.org/10.1111/j.1572-0241.2007.01592.x.

13. Ott SJ, Musfeldt M, Wenderoth DF, Hampe J, Brant O et al. (2004) Reduction in diversity of the colonic mucosa associated bacterial microflora in patients with active inflammatory bowel disease. Gut 53 (5): 685–693.

14. Frank DN, St. Amand AL, Feldman RA, Boedeker EC, Harpaz N et al. (2007) Molecular-phylogenetic characterization of microbial community imbalances in human inflammatory bowel diseases. Proceedings of the National Academy of Sciences 104 (34): 13780–13785. Available: http://www.pnas.org/content/104/34/13780.abstract.

15. Kang S, Denman SE, Morrison M, Yu Z, Dore J et al. (2010) Dysbiosis of fecal microbiota in Crohn's disease patients as revealed by a custom phylogenetic microarray. Inflamm Bowel Dis 16 (12): 2034–2042. Available: http://dx.doi.org/10.1002/ibd.21319.

16. Mukhopadhya I, Hansen R, El-Omar EM, Hold GL (2012) IBD - what role do Proteobacteria play. Nat Rev Gastroenterol Hepatol 9 (4): 219–230.

17. Mondot S, Kang S, Furet JP, Aguirre Carcer D de, McSweeney C et al. (2011) Highlighting new phylogenetic specificities of Crohn's disease microbiota. Inflamm Bowel Dis 17 (1): 185–192. Available: http://dx.doi.org/10.1002/ibd.21436.

18. Joossens M, Huys G, Cnockaert M, Preter V de, Verbeke K et al. (2011) Dysbiosis of the faecal microbiota in patients with Crohn's disease and their unaffected relatives. Gut 60 (5): 631–637.

19. Walker AW, Sanderson JD, Churcher C, Parkes GC, Hudspith BN et al. (2011) High-throughput clone library analysis of the mucosa-associated microbiota reveals dysbiosis and differences between inflamed and non-inflamed regions of the intestine in inflammatory bowel disease. BMC Microbiol 11 (1): 7.

20. Gophna U, Sommerfeld K, Gophna S, Doolittle WF, van Veldhuyzen Zanten SJO (2006) Differences between tissue-associated intestinal microfloras of patients with Crohn's disease and ulcerative colitis. J. Clin. Microbiol. 44 (11): 4136–4141.

21. Cani PD, Possemiers S, van de Wiele T, Guiot Y, Everard A et al. (2009) Changes in gut microbiota control inflammation in obese mice through a mechanism involving GLP-2-driven improvement of gut permeability. Gut 58 (8): 1091–1103.

22. Kato K, Mizuno S, Umesaki Y, Ishii Y, Sugitani M et al. (2004) Randomized placebo-controlled trial assessing the effect of bifidobacteria-fermented milk on active ulcerative colitis. Aliment. Pharmacol. Ther. 20 (10): 1133–1141.

23. Macfarlane S, Furrie E, Cummings JH, Macfarlane GT (2004) Chemotaxonomic analysis of bacterial populations colonizing the rectal mucosa in patients with ulcerative colitis. Clin. Infect. Dis. 38 (12): 1690–1699.

24. Mylonaki M, Rayment NB, Rampton DS, Hudspith BN, Brostoff J (2005) Molecular characterization of rectal mucosa-associated bacterial flora in inflammatory bowel disease. Inflamm. Bowel Dis 11 (5): 481–487.
